# The Transcriptional Corepressor HOS15 Mediates Dark-Induced Leaf Senescence in Arabidopsis

**DOI:** 10.3389/fpls.2022.828264

**Published:** 2022-02-25

**Authors:** Shah Zareen, Akhtar Ali, Chae Jin Lim, Haris Ali Khan, Junghoon Park, Zheng-Yi Xu, Dae-Jin Yun

**Affiliations:** ^1^Department of Biomedical Science and Engineering, Konkuk University, Seoul, South Korea; ^2^Center of Plant Systems Biology and Biotechnology, Plovdiv, Bulgaria; ^3^Key Laboratory of Molecular Epigenetics of the Ministry of Education (MOE), Northeast Normal University, Changchun, China

**Keywords:** HOS15, leaf senescence, developmental aging, dark stress, chromatin remodeling

## Abstract

Multiple endogenous and environmental signals regulate the intricate and highly complex processes driving leaf senescence in plants. A number of genes have been identified in a variety of plant species, including Arabidopsis, which influence leaf senescence. Previously, we have shown that HOS15 is a multifunctional protein that regulates several physiological processes, including plant growth and development under adverse environmental conditions. HOS15 has also been reported to form a chromatin remodeling complex with PWR and HDA9 and to regulate the chromatin structure of numerous genes. However, unlike PWR and HDA9, the involvement of HOS15 in leaf senescence is yet to be identified. Here, we report that HOS15, together with PWR and HDA9, promotes leaf senescence *via* transcriptional regulation of *SAG12/29,* senescence marker genes, and *CAB1/RCBS1A,* photosynthesis-related genes. The expression of *ORE1, SAG12*, and *SAG29* was downregulated in *hos15-2* plants, whereas the expression of photosynthesis-related genes, *CAB1* and *RCBS1A*, was upregulated. HOS15 also promoted senescence through dark stress, as its mutation led to a much greener phenotype than that of the WT. Phenotypes of double and triple mutants of HOS15 with PWR and HDA9 produced phenotypes similar to those of a single *hos15-2*. In line with this observation, the expression levels of *NPX1*, *APG9*, and *WRKY57* were significantly elevated in *hos15-2* and *hos15/pwr, hos15/hda9, and hos15/pwr/hda9* mutants compared to those in the WT. Surprisingly, the total H3 acetylation level decreased in age-dependent manner and under dark stress in WT; however, it remained the same in *hos15-2* plants regardless of dark stress, suggesting that dark-induced deacetylation requires functional HOS15. More interestingly, the promoters of *APG9*, *NPX1*, and *WRKY57* were hyperacetylated in *hos15-2* plants compared to those in WT plants. Our data reveal that HOS15 acts as a positive regulator and works in the same repressor complex with PWR and HDA9 to promote leaf senescence through aging and dark stress by repressing NPX1, APG9, and WRKY57 acetylation.

## Introduction

Leaf senescence is a programmed degeneration process that constitutes the final step of leaf development. Senescence is an organized process regulated by chlorophyll degradation, photosynthesis decline, lipid peroxidation, and protein deprivation (Smart, 1994). The initiation of leaf senescence is a developmentally programmed and apoptotic process that can be controlled through diverse signals, such as environmental factors (including light, nutrients, temperature, and osmotic stress), pathogen attack, and phytohormones (Lim et al., 2007a; Guo and Gan, 2012; Li et al., 2012). To date, several senescence-related mutants and a variety of senescence-associated genes (SAGs), which accumulate during leaf senescence, have been isolated and characterized (Buchanan-Wollaston et al., 2003; Lim et al., 2007a; Li et al., 2012). The expression levels of SAGs increase with leaf aging, while two photosynthetic genes, CHLOROPHYLL A/B BINDING PROTEIN 1 (CAB1) and RIBULOSE BISPHOSPHATE CARBOXYLASE SMALL CHAIN 1A (RBCS1A), have been shown to be downregulated upon aging (Li et al., 2013). In addition, several NAC TFs have been identified in Arabidopsis and crop plants, which play important roles in senescence (Kim et al., 2016; Li et al., 2018). In particular, ANAC016, ANAC002/ATAF1, ANAC029/NAC-LIKE, ANAC019, ANAC032, ACTIVATED BY AP3/PI (NAP), ANAC092/ORESARA1 (ORE1), ANAC046, ANAC055, ANAC057/ORE1 SISTER 1 (ORS1), and ANAC072 promote senescence; ANAC083/VND-INTERACTING2 (VNI2) and ANAC042/JUNGBRUNNEN1 (JUB1) hinder senescence in Arabidopsis (Garapati et al., 2015; Takasaki et al., 2015; Kim et al., 2016; Oda-Yamamizo et al., 2016). The expression levels of NAC genes significantly increase during natural senescence (NS) and artificially induced senescence, such as dark-induced senescence (DIS; Kim et al., 2009, 2013; Sakuraba et al., 2015b). To explore the similarities and variabilities in gene expression levels during senescence and to measure the induction of SAGs by stress, is a major subject of plant researchers (Becker and Apel, 1993; Oh et al., 1996; Chung et al., 1997; Park et al., 1998; Weaver et al., 1998).

High Expression of Osmotically Responsive Genes 15 (HOS15), a WD40 repeat protein, has multiple molecular functions, including the regulation of plant growth and development, cold stress signaling, flowering time determination, abscisic acid (ABA) signaling, response to drought stress, and pathogen response (Zhu et al., 2008; Park et al., 2018a; Ali et al., 2019; Mayer et al., 2019; Park et al., 2019; Shen et al., 2020). During cold stress, HOS15 interacts with and promotes proteasomal degradation of histone deacetylase 2C (HD2C). In addition, it encourages histone 3 (H3) acetylation and keeps “open” the chromatin of cold-responsive (COR) genes and facilitates the recruitment of CBF TFs to the promoter of COR genes for cold stress tolerance (Park et al., 2018a). HOS15 also forms complexes with LUX, ELF3 (evening complex), and HDA9, which bind to the GI promoter and repress the transition to flowering (Park et al., 2019). Furthermore, HOS15 interacts with and degrades OST1, thereby regulating the desensitization of the ABA signaling pathway (Ali et al., 2019). In line with these reports, we have also shown that HOS15 interacts with the SCF-CUL4-E3 ligase complex to repress the plant immune system by negatively regulating NPR1, a pathogen-responsive positive regulator (Shen et al., 2020). Despite all these multiple functions, the role of HOS15 in senescence remains unknown.

In the present study, we report that HOS15 promotes leaf senescence in response to aging and dark stress. Phenotypically, and compared to wild-type (WT) plants, *hos15-2* mutants showed a dramatically late senescence phenotype. While *hos15-2* plants accumulated higher chlorophyll content as well as SAGs were also upregulated in loss-of-function HOS15 mutant plants in relation to WT plants. Moreover, transcript levels of NPX1, APG9, and WRKY57 were upregulated in *hos15-2* compared to WT. Interestingly, compared to WT, the acetylation status of total H3, AcK9, and AcK was higher in *hos15-2*, *pwr*, and *hda9* mutants. Furthermore, we also found a dark impede H3 acetylation level in WT compared *to hos15-2* plants, while the acetylation status of APG9, WRKY57, and NPX1 promoters was also higher in *hos15-2* plants than in WT plants. All these results indicate that HOS15 works in the same complex of PWR and HDA9 to regulate aging and dark-induced leaf senescence through the regulation of the same group of genes.

## Materials and Methods

### Plant Materials

In the present study, the *Arabidopsis thaliana* ecotype Columbia (Col-0) was used as the WT. All the seeds used in the present study were from selected lines, such as Col-0 (WT), *hos15-2*, CL-1, CL-2, *pwr-2,* and *hda9-1*, as described in our previously published research articles (Ali et al., 2019; Baek et al., 2020; Khan et al., 2020). Seeds of the WT, complemented lines, and mutants were surface-sterilized in a solution containing 2% sodium hypochlorite solution (Yakuri Pure Chemicals, Kyoto, Japan) for 5 min and rinsed five times with sterilized water. After stratification for 3 days at 4°C in the dark, sterilized seeds were germinated on full-strength MS medium containing .25% phytagel and 2% sucrose. Ten-day-old seedlings were transferred to the soil under control conditions.

### Growth Conditions

Plants were grown at 23°C under long-day conditions (16-h light/8-h dark photoperiod), under cool white, fluorescent light at a rate of 80–100 μmol m^−2^ s^−1^ in a completely controlled culture room at Konkuk University, Seoul, South Korea. The green rosette and cauline leaves of the selected lines were detached from 4-week-old plants and were then sampled for age-wise leaf senescence phenotype.

### Dark Treatment

For the dark treatment, 4-week-old WT, *hos15-2*, CL-1, and CL-2 plants were exposed to dark stress. However, in terms of leaves, the 1st and 2nd cauline leaves of each ecotype were detached and exposed to 4 days of dark stress. Photographs were taken before and after the dark stress treatment. RNA was extracted from the same leaves, and cDNA was synthesized. Transcript levels were quantified through quantitative real-time PCR (qRT-PCR). In case of nuclear protein extraction to evaluate the histone acetylation status, 12-day-old seedlings of WT and *hos15-2* plants were covered in aluminum foil for 4 days. Nuclear proteins were extracted from stressed and unstressed seedlings using a nuclear protein extraction kit.

### RNA Extraction and qRT-PCR Analysis

For the reverse transcription reactions (PCR), total mRNA (5 μg) was extracted from plants (harvested at different time points for each experiment) using the RNeasy Plant Mini Kit (Qiagen, Hilden, Germany). The RNA was then treated with DNase-1 free Kit (Sigma, St. Louis, MO, United States), and cDNA was synthesized reverse transcription of total RNA using SuperScript III reverse transcriptase (Invitrogen, Carlsbad, CA, United States) with oligo (dT)_12_ primer, according to the manufacturer’s instructions. Quantitative PCR was performed using the SYBR Green PCR Master Mix kit (Bio-Rad, Hercules, CA, United States) according to instructions and using the CFX96 or CFX384 Real-time PCR detection system (Bio-Rad). The PCR mixture (20 μl) comprised 2 μl of first-strand cDNA template, 10 μl of LaboPass™ SYBR Green Q Master (CMQS1000), COSMO GENETECH, South Korea,^1^ and .5 μm of forward and reverse primers for each gene. Three biological replicates were used for each genotype. The relative expression levels were calculated using the comparative cycle threshold method. The sequences of the primers used for the qRT-PCR are listed in Supplementary Table 1. Throughout the study, *ACTIN2* (*ACT2*) was used as the reference to determine relative normalized expression levels during qRT-PCR.

### Chlorophyll Quantification

To measure the total chlorophyll content, frozen leaf tissue was homogenized with zirconia beads, and the pigment was extracted from the leaf homogenate with 80% frozen acetone. The total chlorophyll concentration was determined spectrophotometrically using Biomate 3 (Thermo Electron Corporation, United States), and the optical density (OD) was measured at 663 nm and 645 nm against an 80% acetone blank. The total chlorophyll content was determined using the following equation:


$$\begin{array}{l} \mathrm{Total chlorophyll}\;\left(\mathrm{mg}/g\right)=\left[20.2^{*}{OD}_{645}+8.02^{*}{OD}_{663}\right]\\ \;\times V/1000\times W \end{array}$$

*V* = final volume and *W* = weight of a sample.

### Nuclear Protein Extraction

Tissue samples (.5 g) of 3-week-old seedlings from selected genotypes were sampled in liquid nitrogen and ground manually. The CelLytic PN isolation/extraction Kit (Sigma) was used for nuclear protein extraction as previously described by Wang et al. (2011a).

### Chromatin Immunoprecipitation Assay

Chromatin Immunoprecipitation (ChIP) and ChIP-qRT-PCR were performed according to a previously reported method (Saleh et al., 2008). To fix the chromatin structure, 2-week-old Arabidopsis seedlings were treated with 1% formaldehyde for 15 min and then treated with .1 M glycine for 5 min to stop the cross-linking reaction. The plant tissue was ground with liquid nitrogen, washed with water, and the nuclei were extracted. Nuclear proteins were extracted and sonicated with a Bioruptor (BMS) to fragment the chromosomal DNA. Immunoprecipitation was conducted using the respective antibody, with salmon sperm carrier DNA and Protein-A agarose (Upstate Biotechnology).

### Genetic Crosses

Genetic crosses were performed by transferring mature anthers from the donor to the stigmas of hand-emasculated female recipients.

## Results

### HOS15 Positively Regulates Leaf Senescence

**Figure 1:**
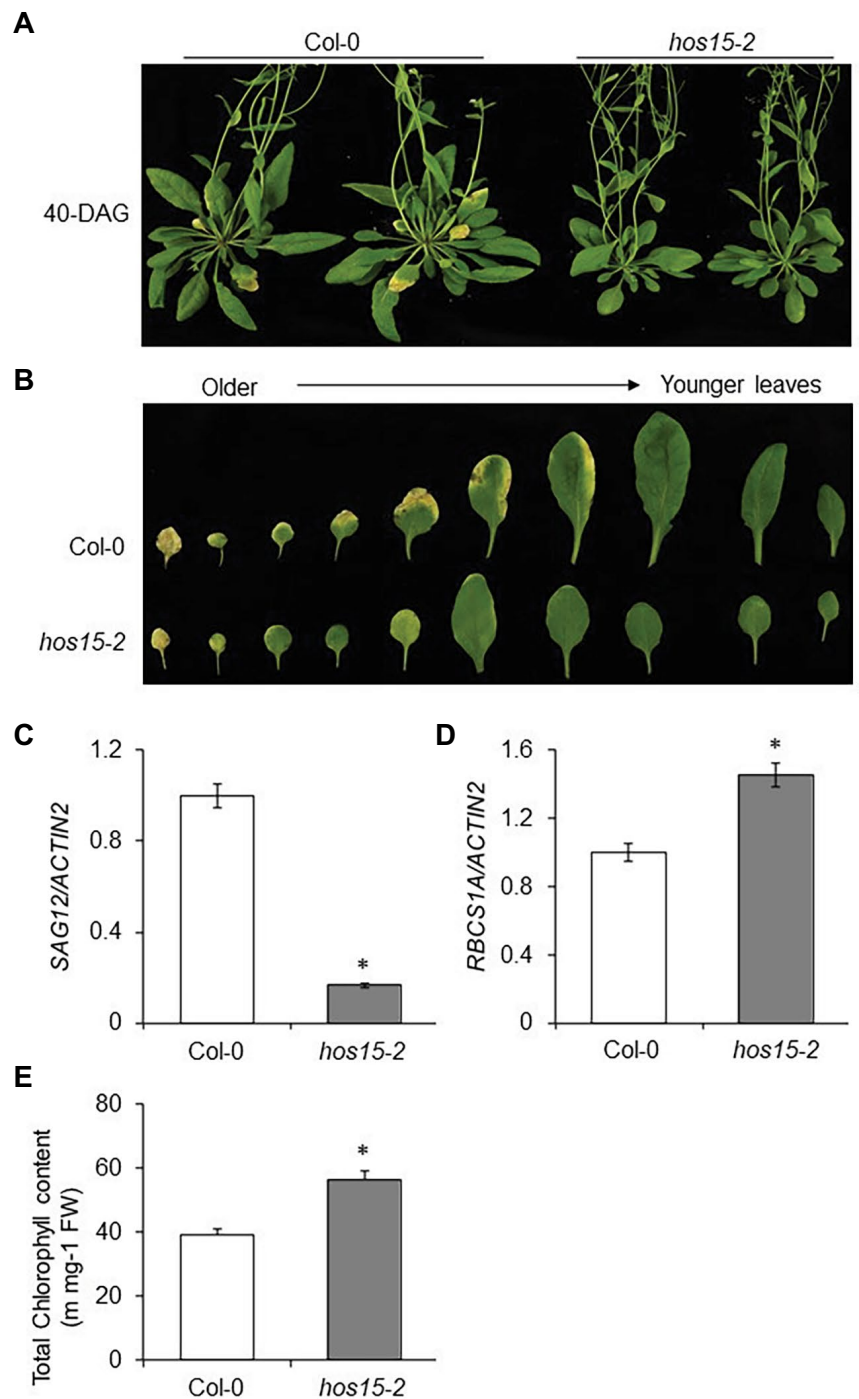
The Arabidopsis *hos15-2* mutant shows late senescence phenotype. **(A)** Comparative phenotypic analysis of Col-0 (WT) and *hos15-2* plants after 40 days of germination. Seeds of Col-0 and *hos15-2* were sterilized and germinated on MS medium for 10 days and then transferred to soil. Photographs were taken after 40 days of germination (DAG). **(B)** Phenotypes of rosette leaves of Col-0 and *hos15-2* plants (3rd to 12th leaves) according to the sorted leaf age of 40 DAG. **(C,D)** Expression of *SAG12*, a senescence marker gene, and *RCBS1A*, a photosynthesis-related gene in 3rd and 4th leaves of Col-0 and *hos15-2* plants. Total RNA was extracted, and qRT-PCR analysis was performed. *ACTIN2* was used as the internal control. Error bars show SD. Differences were determined *via* Student’s *t*-test (significance: ^*^p < .05). **(E)** Leaves of the 4-week-old Col-0 and *hos15-2* plants (3rd and 4th rosette) were used for total chlorophyll content measurement. Error bars show SD. Significant differences were determined *via* Student’s *t*-test (significance: ^*^p < .05).

HOS15 is one of the 85 WD40 repeat proteins in Arabidopsis that function as substrate receptors for the DDB1-CUL4 E3 ligase complex (Lee et al., 2008). Previously, we reported that HOS15 is a multifunctional protein that regulates several physiological processes, including plant development and stress response (Ali and Yun, 2020). However, the involvement of HOS15 in the regulation of leaf senescence is yet to be explored. To investigate the role of HOS15 in leaf senescence, seeds of WT and loss-of-function HOS15 mutant (*hos15-2*) plants were germinated on Murashige and Skoog (MS) plates for 10 days and then transferred to soil. After 40 days of germination, we observed that *hos15-2* mutant plants showed a late senescence phenotype compared to WT and the two complementation lines (Figure 1A; Supplementary Figure 1). As leaf senescence has been considered the final stage of development from maturity to degeneration in the life history of plant leaves (Lim and Hong, 2007), we compared the rosette leaves (3rd to 12th leaves) after 40 days of germination of WT and *hos15-2*. We observed that *hos15-2* rosette leaves were greener than those of WT and the two complementation lines (Figure 1B; Supplementary Figure 1B). These results suggest that HOS15 promotes senescence in Arabidopsis. SAGs have been used as senescence markers because their transcript levels upregulate in an age-dependent (senescence) manner (Li et al., 2013; Sakuraba et al., 2014; Zhang et al., 2015; Ren et al., 2017). The transcript level of *SAG12,* a marker gene, was dramatically reduced in *hos15-2* plants compared to WT plants (Figure 1C; Supplementary Figure 2). Interestingly, *HOS15* was also induced transcriptionally in an age-dependent manner, suggesting the involvement of HOS15 in senescence (Supplementary Figure 2). The transcript level of *RBCS1A* (small subunit of Rubisco 1A), a photosynthesis-related gene, was upregulated in *hos15-2* compared with WT (Figure 1D). In line with the RBCS1A transcript level, the total chlorophyll content also accumulated abundantly in *hos15-2* compared with WT plants (Figure 1E). Taken together, these results demonstrated that HOS15 acts as a positive regulator of leaf senescence.

### HOS15 Regulates Senescence-Associated and Photosynthesis-Related Genes Differentially in an Age-Dependent Manner

**Figure 2:**
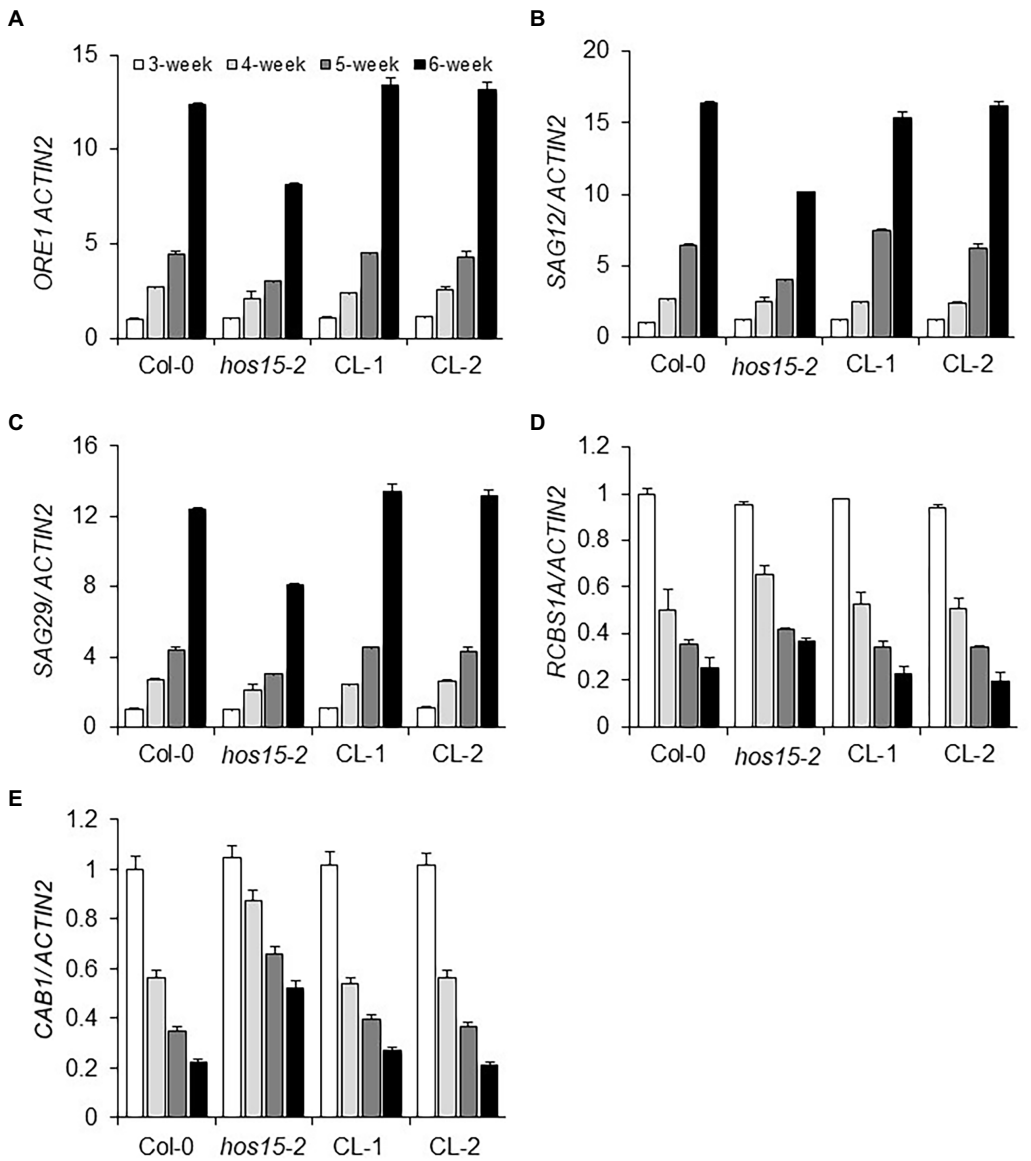
HOS15 differentially regulates senescence- and photosynthesis-related genes in an age-dependent manner. Comparative transcript level analysis of senescence- and photosynthesis-related genes in Col-0 (WT), *hos15-2*, CL-1, and CL-2 plants in an age-dependent manner. **(A–E)** The 3rd and 4th rosette leaves of Col-0, *hos15-2*, CL-1, and CL-2 were sampled at the 3rd, 4th, 5th, and 6th weeks of germination, total RNA was extracted, and cDNA was synthesized. The transcript level of three senescence-related marker genes, *ORE1, SAG12*, and *SAG29*, and two photosynthesis-related genes, *CAB1* and *RBCS1A*, were quantified through qRT-PCR and normalized to 3rd week Col-0. *ACTIN2* was used as the normalization control. Error Bars indicate SD of three independent biological repeats of each sample.

Leaf senescence initiation is a naturally occurring complex process that starts with the upregulation of SAGs and repression of senescence downregulated genes (SDGs; Gepstein et al., 2003; Breeze et al., 2011; Brusslan et al., 2015). A decade ago, Kim et al. (2009) reported that the NAC-type TF family, particularly ORE1/NAC2, promote leaf senescence in an age-dependent manner. In contrast, photosynthesis-related genes, such as *RBCS1A* and *CAB1,* downregulate transcriptionally in an age-dependent manner (Bate et al., 1991; Weaver et al., 1997). To investigate whether HOS15 participates in senescence by regulating both SAGs and photosynthesis-related genes in an age-dependent manner, we analyzed the expression of SAGs in loss-of-function HOS15 mutant and WT plants. In *hos15-2, SAG12*, *SAG29*, and *ORE1* were less expressed in an age-dependent manner, as compared to WT plants (Figures 2A–C). In addition, the transcript levels of photosynthesis-related genes, such as *RCBS1A* and *CAB1,* were significantly higher in *hos15-2* in an age-dependent manner than in WT plants (Figures 2D,E). Taken together, these results suggest that HOS15 regulates senescence in an age-dependent manner through the modulation of senescence- and photosynthesis-related genes.

### Loss-of-Function HOS15 Mutant Delays Leaf Yellowing During Dark-Induced Senescence

**Figure 3:**
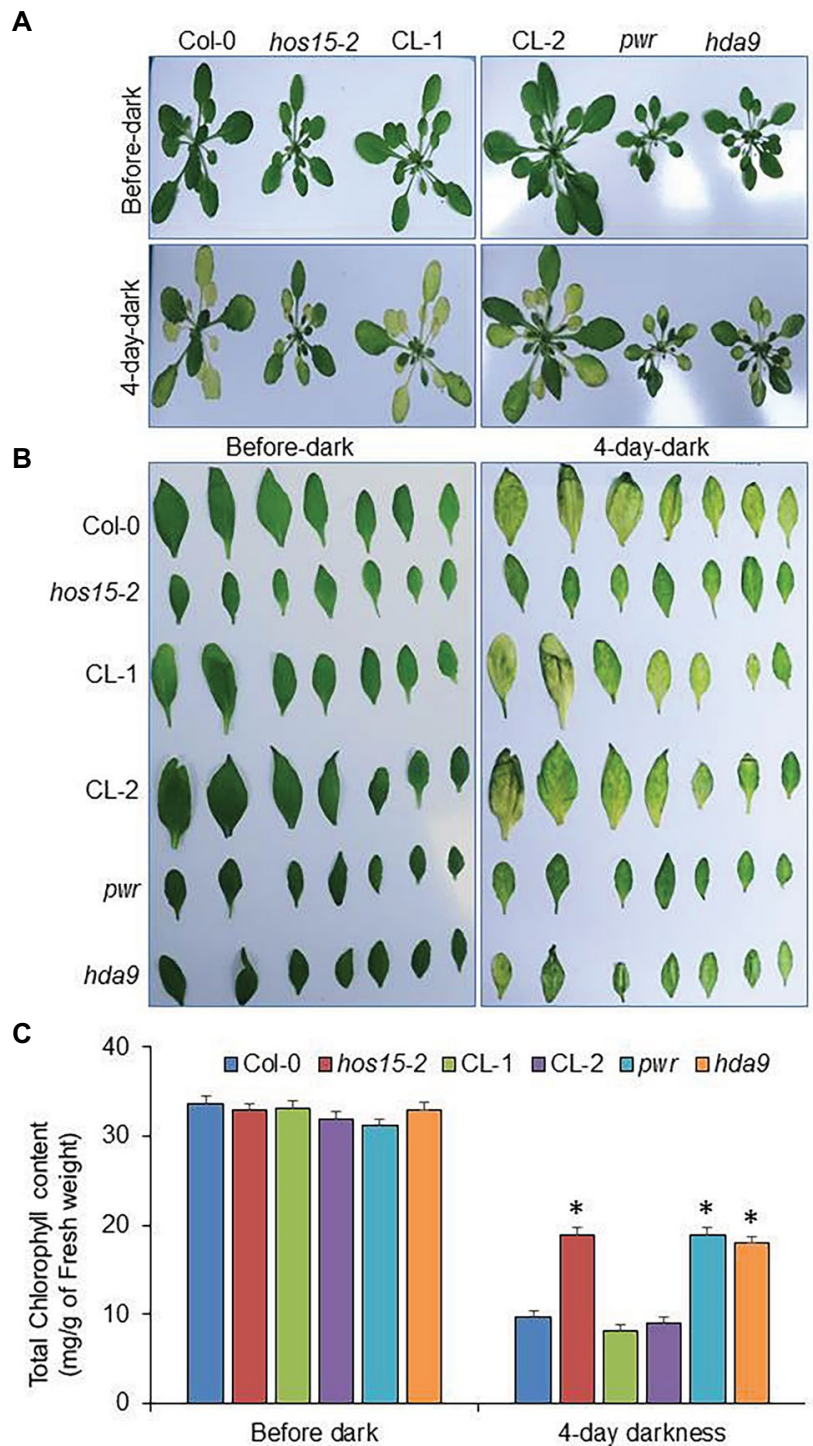
*hos15-2* mutant delays yellowing during dark stress. **(A)** The phenotypic analysis of Col-0 (WT)*, hos15-2*, CL-1, CL-2, *pwr*, and *hda9* plants during dark stress. The 4-week-old plants were treated with dark stress for 4 days. **(B)** Dark-induced phenotype of cauline leaves of Col-0, *hos15-2,* CL-1, CL-2, *pwr*, and *hda9* during dark stress. The cauline leaves (1st and 2nd leaves) of the above-mentioned genotypes were introduced to dark stress for 4 days at room temperature conditions. The photographs were taken before and after dark stress. Each experiment was repeated three times with similar results. **(C)** Leaves of the 4-week-old plants of the indicated genotypes (3rd and 4th rosette) were used for measurement of chlorophyll under control condition and in the presence of 4-day dark stress. Error bars show SD. Significant differences were determined *via* Student’s *t*-test (significance: ^*^p < .05).

All stresses have significant effects on leaf senescence but light privation, either as strong darkening or shading, promotes senescence, particularly when a specific plant part is affected (Weaver and Amasino, 2001; Keech et al., 2010). Several PHYTOCHROME INTERACTING FACTORS (PIFs), a basic helix–loop transcription factor family, including PIF3, PIF4, and PIF5, have been reported to promote natural and dark-induced leaf senescence in Arabidopsis (Trivellini et al., 2012; Sakuraba et al., 2014; Song et al., 2014). In the last decade, dark-induced senescence has been widely used for synchronous promotion and other senescence symptoms, such as chlorophyll degradation (Buchanan-Wollaston et al., 2005). As the PWR-HDA9 complex promotes age-triggered and dark-induced senescence (Chen et al., 2016), we therefore assumed that HOS15 protein may also play a role in dark-induced senescence. We examined the dark-induced senescence phenotype of WT, *hos15-2*, CL-1, CL-2 (CL complementation lines expressing *HOS15::HOS15/hos15-2*), and *pwr* and *hda9,* which were used as positive controls. After 4 days of dark treatment, the *hos15-2, pwr,* and *hda9* plants were greener than WT (Figure 3A). Next, we exposed the detached leaves to dark stress to observe dark-induced senescence, which was also consistent with the plants’ phenotype. The *hos15-2* plant detached leaves were greener than the WT, after 4 days of dark stress (Figure 3B), suggesting that HOS15 plays a positive role in promoting dark-induced senescence in Arabidopsis. Next, we analyzed the total chlorophyll content in leaves under dark stress, as dark stress was shown to impair chlorophyll content (Chen et al., 2016). The similar leaf senescence phenotypes of *hos15-2*, *pwr,* and *hda9* mutants under dark stress were further supported by their similar retention of chlorophyll content (Figure 3C). These observations indicate that HOS15, HDA9, and PWR act in the same pathway to promote leaf senescence.

### HOS15 Differentially Regulates the Expression Pattern of Senescence- and Photosynthesis-Related Genes in Dark Stress

**Figure 4:**
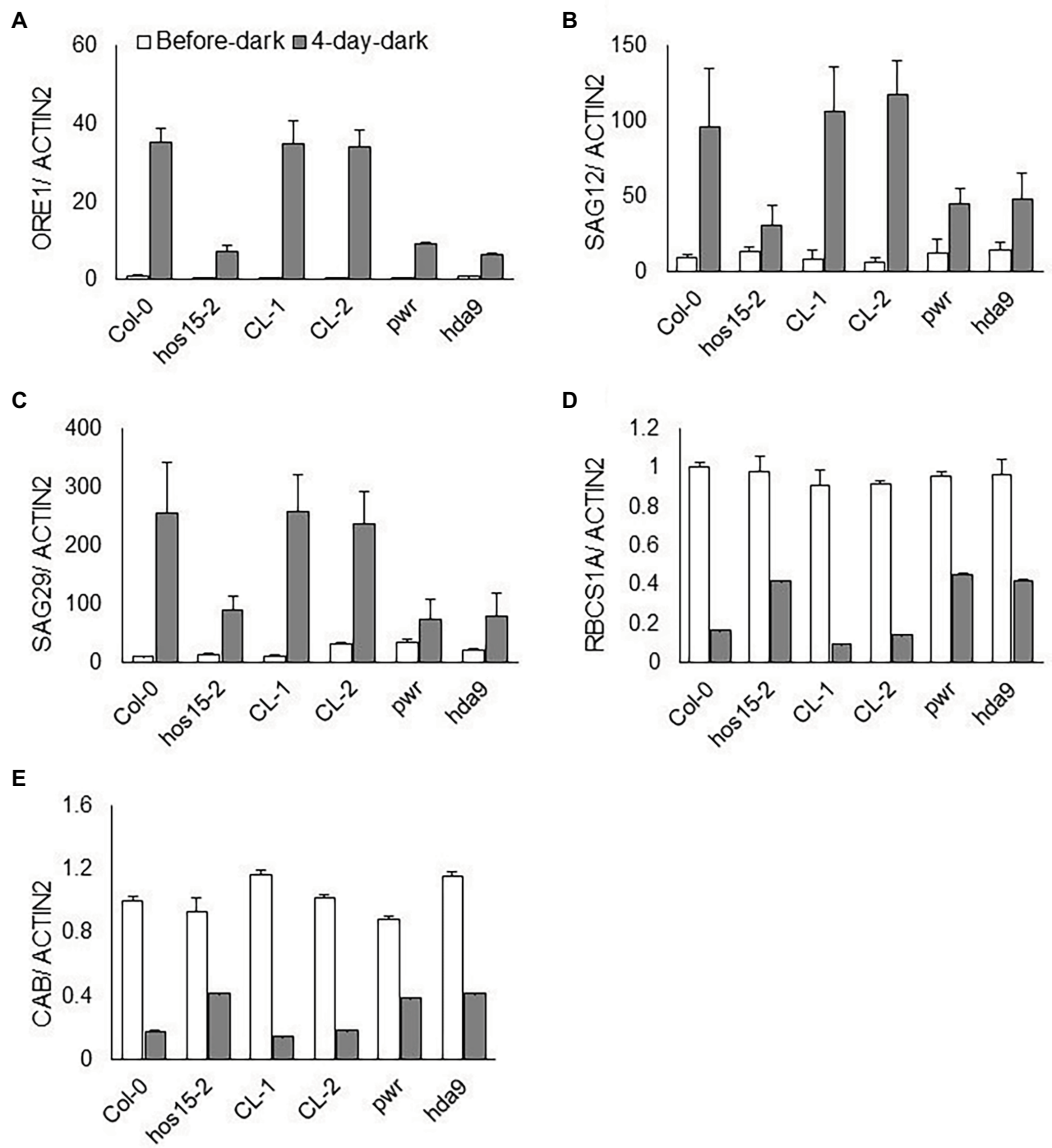
HOS15 differentially regulates senescence- and photosynthesis-related genes during dark-induced senescence. Comparative expression level analysis of senescence- and photosynthesis-related genes. **(A–E)** The transcript level of senescence-related genes *ORE1*, *SAG12*, and *SAG29* and of photosynthesis-related *CAB1* and *RBCS1A* genes before and after dark stress. Cauline leaves of 24-day-old plants were sampled before and after 4 days of dark stress for RNA extraction. The transcript levels were quantified through qRT-PCR and normalized to Col-0 before dark stress. *ACTIN2* was used as the normalization control. Error bars show SD of independent means of three biological repeats.

Dark-induced senescence remarkably accelerates SAG and NAC-type TF gene expression (Chrost et al., 2004). Darkness also reduces photosynthesis efficiency and the expression levels of photosynthesis-related genes (Eckstein et al., 2019). To assess whether HOS15 regulates the transcript abundance of senescence- and photosynthetic-related genes under dark stress, we tested the transcript abundance of senescence-related genes, such as *SAG12, SAG29*, and *ORE1*, and photosynthesis-related genes, such as *CAB1* and *RCBS1A*. Under dark stress, the transcript levels of *SAG12, SAG29*, and *ORE1* were dramatically reduced in *hos15-2* compared to WT plants (Figures 4A–C). In contrast, *CAB1* and *RCBS1A* were dramatically upregulated in *hos15-2* plants compared to WT under dark stress (Figures 4D,E). These findings suggest that HOS15 promotes dark-induced senescence through the differential regulation of senescence- and photosynthesis-related genes.

### HOS15 Together With PWR-HDA9 Complex Co-regulates Senescence

**Figure 5:**
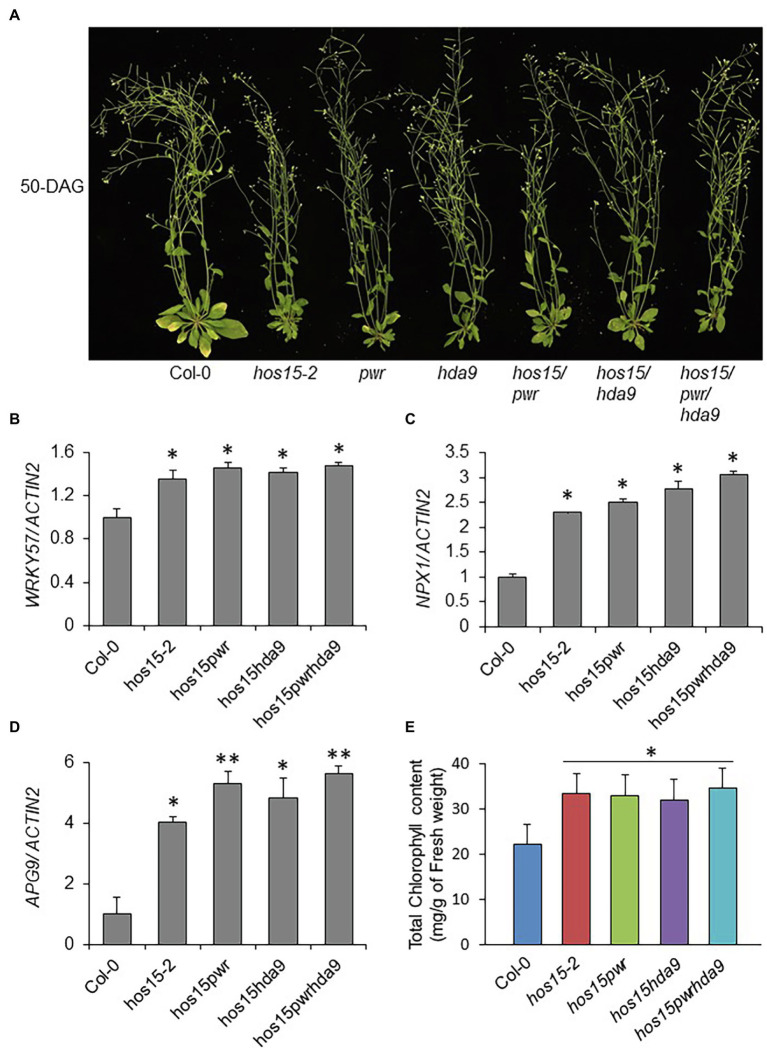
HOS15-PWR-HDA9 complex promotes senescence through the regulation of same group genes. **(A)** Loss-of-function of HOS15-PWR-HDA9 complex components and its double and triple mutants showing late senescence phenotype after 50 days of germination. Seeds of Col-0, *hos15-2, pwr, hda9, hos15pwr, hos15hda9*, and *hos15pwrhda9* plants were grown on MS-media for 12 days and then transferred to soil. **(B–D)** The transcript analysis of senescence negative regulator genes, *WRKY57, NPX1,* and *APG9* after 4 weeks of germination. Total RNA was extracted from the indicated genotypes and cDNAs were synthesized. The expression level was quantified using qRT-PCR. *ACTIN2* was used as the internal control. Error bars represent SD from three biological replicates. Significant differences were determined *via* Student’s *t*-test (significance: ^*^p < .05, ^**^p < .01). **(E)** Leaves of the 4-week-old plants of the indicated genotypes (3rd and 4th rosette) were used for measurement of total chlorophyll content. Error bars show SD. Significant differences were determined *via* Student’s *t*-test (significance: ^*^p < .05).

Recently, the PWR-HDA9 complex has been shown to regulate leaf senescence, as their mutations cause age-triggered and dark-induced senescence phenotypes (Chen et al., 2016). HOS15 has also been reported to interact with and work in the same complex with HDA9-PWR to regulate plant growth and development (Park et al., 2018b; Mayer et al., 2019). To test the genetic relationship between HOS15 and the HDA9-PWR complex in the regulation of plant leaf senescence, we generated double and triple mutants (*hos15pwr*, *hos15hda9*, and *hos15pwrhda9*). As expected, the loss-of-function HOS15, PWR, and HDA9 individually, and their double and triple mutants (*hos15pwr*, *hos15hda9*, and *hos15pwrhda9*) showed late senescence phenotypes compared to WT plants (Figure 5A). It has been shown that PWR recruits HDA9 to W-Box-containing genes, *APG9* (autophagy), *WRKY57* (jasmonic acid), and *NPX1* (ABA catabolism), which negatively regulate their transcription and promote senescence (Chen et al., 2016). To investigate whether HOS15 also regulates the same group of genes during senescence, we evaluated the expression levels of *NPX1, APG9*, and *WRKY57*. Compared to WT, transcript levels of *NPX1, APG9*, and *WRKY57* genes were significantly upregulated in *hos15-2*, *hos15pwr*, *hos15hda9*, and *hos15pwrhda9* mutants (Figures 5B–D). Late senescence phenotypes of *hos15-2*, *pwr*, *hda9*, *hos15/pwr*, *hos15/hda9* and *hos15/pwr/hda9* mutants were further supported by their similar retention of chlorophyll content (Figure 5E). These results demonstrated that HOS15, PWR, and HAD9 might work together in the same complex to regulate leaf senescence by regulating the same group of genes.

### Regulation of H3 Acetylation Status Is Essential for Age-Dependent Leaf Senescence

Previous reports have shown that PWR-HDA9 modulates H3 acetylation status and that their mutation results in increased H3 acetylation levels (Chen et al., 2016; Khan et al., 2020; Lim et al., 2020). Similarly, HOS15 is also involved in the regulation of H3 acetylation status through different signaling pathways (Park et al., 2018a; Mayer et al., 2019). To investigate the combined role of this co-repressor complex in histone modulation, we assessed H3 acetylation status in *hos15, hda9,* and *pwr* (single, double, and triple) mutants. As expected, the acetylation levels of H3 (AcH3), H3K9 (AcH3K9), and acetylated lysine (AcK) were remarkably induced in *hos15-2, pwr, hda9, hos15/pwr, hos15/hda9*, and *hos15/pwr/hda9* mutants as compared to WT plants, suggesting that the HOS15-PWR-HDA9 complex represses H3 acetylation (Supplementary Figure 3A).

**Figure 6:**
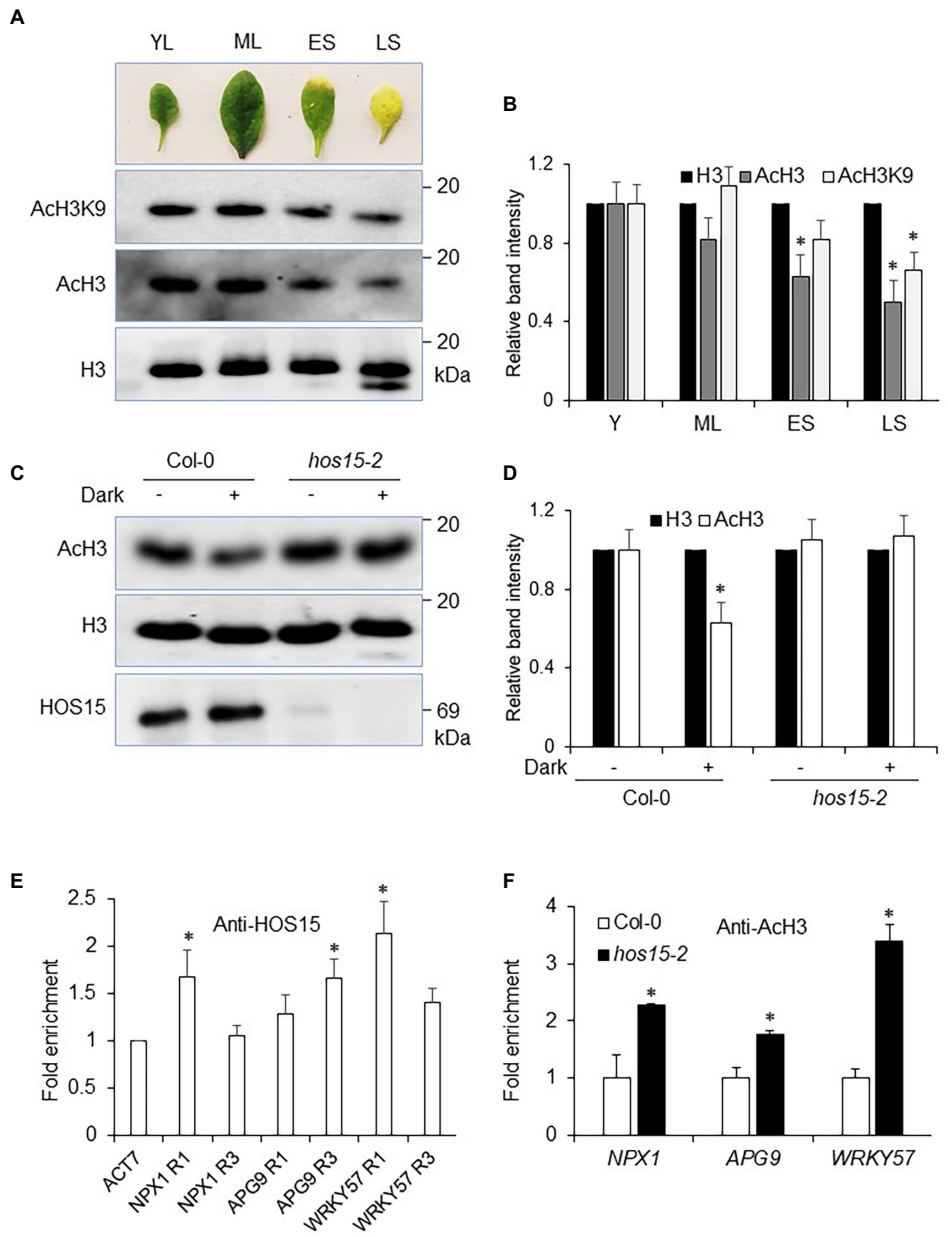
HOS15 represses H3 acetylation status during senescence. **(A)** Plant senescence/aging negatively regulates histone acetylation status. Same age leaves were sampled for nuclear protein extraction. Anti-H3, AcH3, and AcH3K9 antibodies were used to detect the acetylation level in leaves in an age-dependent manner (YL = young leaves, ML = mature leaves, ES = early senescence, and LS = late senescence leaves; previously described by Sun et al., 2017). **(B)** Relative band intensity of H3, AcH3, and AcH3K9 in (A), quantified by ImageJ software, with signal level of H3 set to 1. Error bars represent SE. Significant differences were determined *via* Student’s *t*-test (significance: ^*^p < .05). **(C)** HOS15 regulates dark-induced senescence by affecting H3 acetylation status. Twelve-day-old seedlings of Col-0 and *hos15-2* plants were treated with 4 days of darkness, and then, nuclear proteins were extracted. H3, AcH3 and HOS15 antibodies were used for WB. H3 was used as the loading control. **(D)** Relative band intensity of H3 and AcH3 in **(C)**, quantified by ImageJ software, with signal level of H3 set to 1. Error bars represent SE. Significant differences were determined *via* Student’s *t*-test (significance: ^*^p < .05). **(E)** ChIP assay was performed with anti-HOS15 antibody in Col-0 (WT) plants. *hos15-2* plants were used as negative control. The amount of DNA in the immunoprecipitated complex was determined by RT-qPCR and is presented as the fold enrichment after normalization with control using the ACTIN7 gene promoter. Data represent means (SD) from three biological replicates with three technical repeats. Significant differences were determined *via* Student’s *t*-test (significance: ^*^p < .05). Specific loci used for ChIP assay are described in Supplementary Figure 4. **(F)** Chip-qRT-PCR for the leaf senescence negative regulator genes; *NPX1, APG9*, and *WRKY57.* H3 acetylation status were increased in *hos15-2* as compared to Col-0 plants. *ACTIN2* was used as the normalization control. Error bars represent SD of three biological repeats. Significant differences were determined *via* Student’s *t*-test (significance: ^*^p < .05).

Leaf aging and environmental stresses have been considered to play a dynamic role in plant senescence regulation. However, how aging and environmental stresses, particularly dark stress, regulate histone acetylation status remains elusive. Recently, JMJ16, an Arabidopsis JmjC domain-containing protein and H3K4-specific demethylase, was found to repress age-dependent plant leaf senescence through its demethylase function (Liu et al., 2019). To elucidate the effect of leaf aging on histone acetylation status, we determined the H3 acetylation status in an age-dependent manner (YL = young leaves, ML = mature leaves, ES = early senescence, and LS = late senescence; Previously shown by). We found that age triggered a dramatic reduction in H3 acetylation status, particularly H3K9. For instance, acetylation levels in YL and ML were abundantly accumulated as compared to LS leaves (Figures 6A,B), suggesting that plant aging (senescence) reduced H3 acetylation status to promote leaf senescence.

### HOS15 Negatively Regulates H3 Acetylation Status to Promote Dark-Induced Senescence

Recently, high light (HL) was found to increase the acetylation status of H3 (Guo et al., 2008). As *hos15-2* shows delayed senescence under dark stress, we were interested in exploring the functional role of HOS15 in the modulation of H3 acetylation level during dark stress. As expected, dark stress significantly reduced H3 acetylation in WT (Figures 6C,D). In contrast, H3 acetylation levels remained highly stable in *hos15-2* single mutant, *hos15-2/hda9* and *hos15-2/pwr* double mutants, and *hos15-2/hda9/pwr* triple mutant plants regardless of dark stress, suggesting that dark-induced deacetylation requires functional HOS15, HDA9, and PWR (Figures 6C,D; Supplementary Figure 3B).

According to Chen et al. (2016), PWR and HDA9 directly repress the acetylation status of *NPX1, APG9*, and *WRKY57* to promote leaf senescence. In this regard, we were interested in examining the role of HOS15 in the regulation of *NPX1*, *APG9*, and *WRKY57*, as the expression levels of these genes were found to be dramatically higher in *hos15-2* plants than in WT (Figure 5B). We first tested the direct association of HOS15 with the promoters of *NPX1*, *APG9,* and *WRKY57* and found that HOS15 associates with the promoters of these genes at specific regions which were previously identified as target loci for PWR and HDA9 (Figure 6E; Supplementary Figure 4; Chen et al., 2016). Next, we analyzed H3 acetylation level on these specific regions using Anti-AcH3 antibodies. As shown in Figure 6F, the promoters of *NPX1, APG9*, and *WRKY57* were hyper-acetylated in loss-of-function HOS15 mutant as compared with WT plants. Taken together, these findings demonstrate that HOS15 regulates senescence by repressing the acetylation levels of *NPX1, APG9*, and *WRKY57* and that HOS15 works together with PWR-HDA9 in the same complex to regulate aging and dark-induced leaf senescence.

## Discussion

### HOS15, a WD40 Domain Protein, Regulates Senescence Through Aging

HOS15 acts as a multifunctional protein, and it is actively involved in the regulation of plant growth, development, and stress response. In recent years, we have reported the involvement of HOS15 in cold stress, flowering transition, ABA signaling, drought stress, and pathogen responses (Zhu et al., 2008; Park et al., 2018a; Ali et al., 2019; Mayer et al., 2019; Park et al., 2019; Shen et al., 2020). Interestingly, Mayer et al. (2019) reported that cell division and light-responsive genes were downregulated in the *hda9/hos15* double mutant. Here, we report that HOS15 is also involved in the regulation of senescence through aging and darkness. *hos15-2* plants showed a late senescence phenotype compared to WT after 40 days of germination (Figure 1). Aside from exogenous stresses, senescence generally depends on the leaf age and developmental stage (Zentgraf et al., 2004). We also found that *hos15-2* showed a late leaf senescence phenotype compared to WT (Figure 1B). Due to senescence, expression levels of some genes were classified into up- and downregulated; SAGs and senescence downregulated genes (SDGs), respectively (Lohman et al., 1994). In this diverse network, we chose two upregulated SAGs, SAG12, and SAG29, as senescence marker genes. Similar to PWR and HDA9, HOS15 also positively regulated the SAG12/29 transcript levels (Chen et al., 2016; Figure 2), suggesting that HOS15 plays a positive role in the regulation of these markers during senescence. We found that the expression levels of SAG12 and SAG29 were significantly lower in *hos15-2* than in WT plants after 40 days of germination (Figure 2). Moreover, HOS15 also plays a negative role in the regulation of total chlorophyll content in plants, as the total chlorophyll content was found to be much higher in *hos15-2* plants than in WT plants (Figure 1E). Taken together, these results indicate that HOS15 acts as a positive regulator of leaf senescence.

Leaf senescence is also involved in chromatin modification, which leads to changes in gene expression patterns. Natural leaf senescence is mainly recognized by aging, which can be triggered by environmental cues and nutritional signals, such as phytohormones, oxidants, abiotic and biotic stresses, and darkness (Mayta et al., 2019). PWR-HDA9-HOS15 work together in the same suppressor complex to regulate plant development and morphological processes (Mayer et al., 2019). Several genes have been identified and reported to be involved in the regulation of plant senescence in an age-dependent manner. Interestingly, PWR and HDA9 promote senescence through aging (Chen et al., 2016), and we expected that HOS15 would be involved in senescence. We found that the expression of HOS15 was gradually induced by age in the 3rd and 4th rosette leaves (Supplementary Figures 2A,B). We also evaluated the transcript level of the senescence marker gene *SAG12*, which was less expressed in *hos15-2*, compared to WT, in an age-dependent manner (Supplementary Figures 2A,B). We observed that the SAG12 transcript level was low and appeared almost 2 weeks later in *hos15-2*, as SAG12 mRNA upregulation appeared 4 weeks after germination in WT and 6 weeks after germination in *hos15-2* (Figure 2A). This evidenced that HOS15 participates as an activator of leaf senescence in an age-dependent manner. In line with this, *hos15-2* showed a late senescence phenotype after 35, 45, and 60 days of germination, respectively, compared to WT (Supplementary Figure 1). Like other senescence events in plants, leaf senescence is the final stage of development and decadence from maturation in the history of leaf life (Lim and Hong, 2007). Overall, *hos15-2* rosette leaves of increasing age (older to younger) were greener than that of WT plants (Supplementary Figure 1). Hence, based on phenotypes, we showed that HOS15 plays a positive role in leaf senescence regulation through the aging process. In addition, the NAC-type TF family plays an important role in promoting leaf senescence. Kim et al. (2009) reported that *ore1* delayed senescence by aging. In the HOS15 loss-of-function mutant, the ORE1 transcript level was significantly downregulated compared to that in the WT in an age-dependent manner (Figure 2). RCBS1A and CAB1 play crucial roles during photosynthesis and CO_2_ fixation (Izumi et al., 2012; Bresson et al., 2018). The expression levels of *RCBS1A* and *CAB1* were significantly elevated in the HOS15 loss-of-function mutant compared to those in WT in an age-dependent manner (Figure 2). Taken together, these phenotypical and genetic assessments revealed that HOS15 positively regulates senescence in an age-dependent manner, promotes the expression of SAGs, and suppresses that of photosynthesis-related genes.

### HOS15 Acts as Senescence Inducer in Dark Stress

Leaf senescence can also be induced by several exogenous factors, such as dark stress (Lim and Hong, 2007). Interestingly, HAD9 and PWR induce dark-induced leaf senescence (Chen et al., 2016); therefore, we hypothesized that HOS15 might also play a positive role in the regulation of dark-induced leaf senescence. We scrutinized the detached leaf yellowing and found that leaf yellowing was attenuated in *hos15-2* as compared to WT, after 4 days of dark treatment (Figure 3), suggesting that HOS15 acts as a positive regulator in dark-induced senescence. Furthermore, in response to 4 days of darkness, the transcript levels of SAG12/29 and ORE1 were significantly less induced in the loss-of-function HOS15 mutant than in the WT (Figures 4A–C). In contrast, the expression levels of *RCBS1A* and *CAB1*, photosynthesis-related genes, were higher in *hos15-2* than in WT under dark stress (Figures 4D,E). Taken together, these data suggest that HOS15 promotes dark-induced leaf senescence by positively regulating senescence-related genes and negatively regulating photosynthesis-related genes.

### HOS15-PWR-HDA9 Complex Regulates the Same Group of Genes Involved in Senescence

HOS15, PWR, and HDA9 have been reported to be predominantly localized in the nucleus. Particularly, PWR has been shown to be involved in the promotion of several micro-RNA genes, which are involved in the regulation of senescence. In addition, a number of ABA signaling (Gao et al., 2016), oxidative stress (Du et al., 2008), jasmonic acid (Jiang et al., 2014), salicylic acid (Veronese et al., 2006), and autophagy pathway (Wang et al., 2011b) genes have been reported to mediate the regulation of plant senescence (Chen et al., 2016). It has also been shown that PWR and HDA9 are involved in leaf senescence through regulation of *APG9, NPX1,* and *WRKY57* genes (Chen et al., 2016). We found that *NPX1, APG9,* and *WRKY57* were significantly upregulated in *hos15-2*, *hos15/pwr, had15/hda9*, and *hhad5/pwr/hda9* mutants as compared to WT (Figure 5A), suggesting that HOS15 and PWR-HDA9 work in the same complex to promote leaf senescence in Arabidopsis.

### HOS15 Promotes Age-Dependent and Dark-Induced Senescence Through Regulation of Chromatin Structure of NPX1, APG9, and WRKY57

Histone acetylation is often associated with active transcription and open chromatin, whereas deacetylation is generally considered an inactive and compact structure to repress transcription (Verdin and Ott, 2015). PWR-HDA9 modulates the H3 acetylation status and its mutation results in increased acetylation levels (Chen et al., 2016; Khan et al., 2020; Lim et al., 2020). Similarly, HOS15 is also involved in the regulation of histone status through different signaling pathways (Mayer et al., 2019; Park et al., 2019). The physical association of HOS15with PWR and HDA9 led us to investigate the role of HOS15 in the regulation of histone status (acetylation/deacetylation; Mayer et al., 2019; Lim et al., 2020). We found that the acetylation levels of H3 (AcH3), H3K9 (AcH3K9), and acetylated lysine (AcK) were remarkably increased in *hos15-2, pwr, hda9,* and their double and triple mutants compared to WT (Supplementary Figure 3A). These observations suggest that the HOS15-PWR-HDA9 complex represses histone acetylation. Leaf aging and dark stress play a vital role in the regulation of leaf senescence. To elucidate the effect of leaf aging on histone acetylation status, we assessed the acetylation level of H3, which was dramatically reduced in late senescence as compared to young leaves (Figure 6A). These findings suggest that aging decreases H3 acetylation levels and encourages senescence. In contrast, HL increases the H3 acetylation level (Guo et al., 2008). In this study, we observed that unlike HL, dark stress decreases H3 acetylation level in WT, which is largely controlled by HOS15, HDA9, and PWR (Figures 6C,D; Supplementary Figure 3B), suggesting that HOS15 together with HDA9 and PWR negatively regulates H3 acetylation levels under dark stress to enhance senescence. HOS15 was further found to repress the acetylation status of *NPX1, APG9*, and *WRKY57* promoters to promote leaf senescence. These results strongly support the notion that like HDA9 and PWR, HOS15 also regulates senescence through repression of the same group of genes. Taken together, our results suggest that HOS15 together with the PWR-HDA9 complex regulates aging and dark-induced leaf senescence by modulating histone acetylation status on the promoters of *NPX1, APG9,* and *WRKY57*. Even though the total H3 acetylation level decreases upon senescence (Figure 6A) as well as under dark stress (Figure 6C; Supplementary Figure 3B), H3 acetylation level at the chromatin of specific genes, such as SAG genes, might increases during senescence. According to previous reports, total H3 acetylation level increases in *hos15-2*, *pwr,* and *hda9* mutants (Chen et al., 2016; Mayer et al., 2019). However, recently Lim et al. (2020) reported that even though the total H3 acetylation level increases in *hos15*-*2* and *pwr* mutants (Mayer et al., 2019), acetylation level at the promoters of specific genes decreases in these mutants. For instance, compared to WT, H3 acetylation level at the promoter of *COR15A* decreases in *hos15* and *pwr* mutants (Lim et al., 2020). These observations suggest that change in total H3 acetylation level is different from H3 acetylation level at certain chromatins. Decrease in H3 acetylation level upon senescence and dark stress represent major goals for future studies.

## Proposed Model

**Figure 7:**
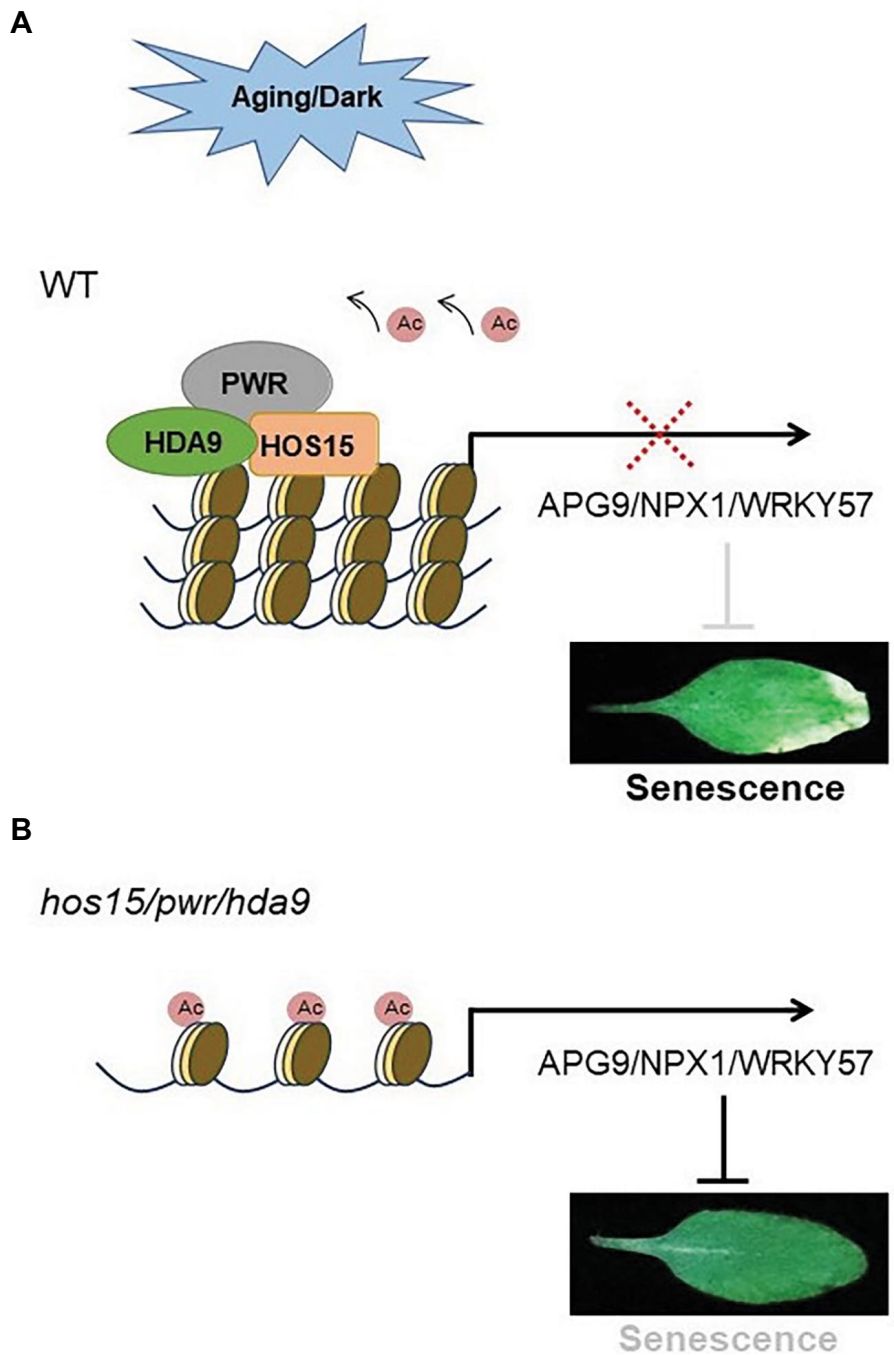
Proposed model. **(A)** *HOS15* works with HDA9/PWR in the same complex, which positively regulates plant senescence through developmental aging process and dark stress. In the developmental aging process or dark stress, the HOS15-PWR-HDA9 complex promotes senescence through the repression of the same target genes, such as *NPX1, APG9*, and *WRKY57*, which are the key negative regulators of plant senescence. **(B)** *NPX1, APG9*, and *WRKY57* are hyperacetylated in plants that lack the HOS15/PWR/HDA9 complex as a whole or any component of this complex. Hyperacetylation of *NPX1, APG9,* and *WRKY57* leads to strong inhibition of age-dependent or dark-induced senescence.

Plant developmental aging and dark stress are the two main factors that coordinately regulate leaf senescence. In Arabidopsis, HOS15-PWR-HDA9 work in the same corepressor complex to repress a wide range of genes (Figure 7). In our proposed model, HOS15 also acts as a senescence inducer through developmental aging and dark stress, working together with PWR and HDA9 (Figure 7). During the developmental aging process and dark stress, HOS15 negatively regulates the acetylation status of the same group of genes, such as *APG9, NPX1*, and *WRKY57* (senescence negative regulators) to modulate plant senescence in Arabidopsis.

## Data Availability Statement

The original contributions presented in the study are included in the article/Supplementary Material, further inquiries can be directed to the corresponding author.

## Author Contributions

SZ, AA, and D-JY conceived and designed the experiments, analyzed the data, and wrote the paper. SZ, AA, HK, JP, CL, and Z-YX performed the experiments. All authors contributed to the article and approved the submitted version.

## Funding

This work was supported by grants from the National Research Foundation of Korea (NRF), Global Research Lab (2017K1A1A2013146), National Research Foundation of Korea (NRF) funded by the Korean Government (2019R1A2C2084096 to D-JY) and the Next-Generation Bio-Green21 Program of Rural Development Administration (RDA), Republic of Korea (PJ015968 to D-JY).

## Conflict of Interest

The authors declare that the research was conducted in the absence of any commercial or financial relationships that could be construed as a potential conflict of interest.

## Publisher’s Note

All claims expressed in this article are solely those of the authors and do not necessarily represent those of their affiliated organizations, or those of the publisher, the editors and the reviewers. Any product that may be evaluated in this article, or claim that may be made by its manufacturer, is not guaranteed or endorsed by the publisher.

## Footnotes

^1^ http://www.cosmogenetech.com/co.kr

## References

[ref1] Ali A. Kim J. K. Jan M. Khan H. A. Khan I. U. Shen M. Z.. (2019). Rheostatic control of ABA signaling through HOS15-mediated OST1 degradation. Mol. Plant 12, 1447–1462. doi: 10.1016/j.molp.2019.08.005, PMID: 31491477

[ref2] Ali A. Yun D. J. (2020). Arabidopsis HOS15 is a multifunctional protein that negatively regulate ABA-signaling and drought stress. Plant Biotechnol. Rep. 14, 163–167. doi: 10.1007/s11816-020-00600-1

[ref3] Baek D. Shin G. Kim M. C. Shen M. Lee S. Y. Yun D.-J. (2020). Histone Deacetylase HDA9 With ABI4 contributes to Abscisic acid homeostasis in drought stress response. Front. Plant Sci. 11:143. doi: 10.3389/fpls.2020.00143, PMID: 32158458 PMC7052305

[ref4] Bate N. J. Rothstein S. J. Thompson J. E. (1991). Expression of nuclear and chloroplast photosynthesis-specific genes during leaf senescence. J. Exp. Bot. 42, 801–811. doi: 10.1093/jxb/42.6.801

[ref5] Becker W. Apel K. (1993). Differences in gene expression between natural and artificially induced leaf senescence. Planta 189, 74–79. doi: 10.1007/BF00201346

[ref6] Breeze E. Harrison E. McHattie S. Hughes L. Hickman R. Hill C.. (2011). Highresolution temporal profiling of transcripts during Arabidopsis leaf senescence reveals a distinct chronology of processes and regulation. Plant Cell 23, 873–894. doi: 10.1105/tpc.111.083345, PMID: 21447789 PMC3082270

[ref7] Bresson J. Stefan B. Lena R. Jasmin D. Ulrike Z. (2018). A guideline for leaf senescence analyses: from quantification to physiological and molecular investigations. J. Exp. Bot. 69, 769–786. doi: 10.1093/jxb/erx246, PMID: 28992225

[ref8] Brusslan J. A. Bonora G. Rus-Canterbury A. M. Tariq F. Jaroszewicz A. Pellegrini M. (2015). A genome-wide chronological study of gene expression and two histone modifications, H3K4me3 and H3K9ac, during developmental leaf senescence. Plant Physiol. 168, 1246–1261. doi: 10.1104/pp.114.252999, PMID: 25802367 PMC4528724

[ref9] Buchanan-Wollaston V. Earl S. Harrison E. Mathas E. Navabpour S. Page T.. (2003). The molecular analysis of leaf senescence-A genomics approach. Plant Biotechnol. J. 1, 3–22. doi: 10.1046/j.1467-7652.2003.00004.x, PMID: 17147676

[ref10] Buchanan-Wollaston V. Tania P. Elizabeth H. Emily B. Pyung O. L. Hong G. N.. (2005). Comparative transcriptome analysis reveals significant differences in gene expression and signaling pathways between developmental and dark/starvation-induced senescence in Arabidopsis. Plant J. 42, 567–585. doi: 10.1111/j.1365-313X.2005.02399.x, PMID: 15860015

[ref11] Chen X. Li L. Kevin S. M. Mark S. Shuiming Q. Aaron L.. (2016). POWERDRESS interacts with HISTONE DEACETYLASE 9 to promote aging in Arabidopsis. eLife 5:e17214. doi: 10.7554/eLife.17214, PMID: 27873573 PMC5119886

[ref12] Chrost B. Daniel A. Krupinska K. (2004). Regulation of alpha-galactosidase gene expression in primary foliage leaves of barley (*Hordeum vulgare* L.) during dark-induced senescence. Planta 218, 886–889. doi: 10.1007/s00425-003-1166-5, PMID: 14652758

[ref13] Chung B. C. Lee S. Y. Oh S. A. Rhew T. H. Nam H. G. Lee C. H. (1997). The promoter activity of sen1, a senescence-associated gene of Arabidopsis, is repressed by sugars. J. Plant Physiol. 151, 339–345. doi: 10.1016/S0176-1617(97)80262-3

[ref14] Du Y. Y. Wang P. C. Chen J. Song C. P. (2008). Comprehensive functional analysis of the catalase gene family in Arabidopsis thaliana. J. Integr. Plant Biol. 50, 1318–1326. doi: 10.1111/j.1744-7909.2008.00741.x, PMID: 19017119

[ref15] Eckstein A. Joanna G. Pawel H. Justyna L. Agnieszka K. B. (2019). A role for GLABRA1 in dark-induced senescence. Acta Biochim. Pol. 66, 243–248. doi: 10.18388/abp.2018_2825, PMID: 31254977

[ref16] Gao S. Gao J. Zhu X. Song Y. Li Z. Ren G.. (2016). ABF2, ABF3, and ABF4 promote ABA-mediated chlorophyll degradation and leaf senescence by transcriptional activation of chlorophyll catabolic genes and senescence-associated genes in Arabidopsis. Mol. Plant 9, 1272–1285. doi: 10.1016/j.molp.2016.06.006, PMID: 27373216

[ref17] Gepstein S. Sabehi G. Carp M. J. Hajouj T. Nesher M. F. Yariv I.. (2003). Large-scale identification of leaf senescence-associated genes. Plant J. 36, 629–642. doi: 10.1046/j.1365-313X.2003.01908.x, PMID: 14617064

[ref200] Garapati P. Xue G. P. Munné-Bosch S. Balazadeh S. (2015). Transcription Factor ATAF1 in Arabidopsis Promotes Senescence by Direct Regulation of Key Chloroplast Maintenance and Senescence Transcriptional Cascades. Plant Physiol. 168, 1122–39. doi: 10.1104/pp.15.00567, PMID: 25953103 PMC4741325

[ref18] Guo Y. Gan S. S. (2012). Convergence and divergence in gene expression profiles induced by leaf senescence and 27 senescence promoting hormonal, pathological and environmental stress treatments. Plant Cell Environ. 35, 644–655. doi: 10.1111/j.1365-3040.2011.02442.x, PMID: 21988545

[ref400] Guo L. Zhou J. Elling A. A. Charron J.-B. F. Deng X. W. (2008). Histone Modifications and Expression of Light-Regulated Genes in Arabidopsis Are Cooperatively Influenced by Changing Light Conditions. Plant Physiol. 147, 2070–83. doi: 10.1104/pp.108.122929, PMID: 18550682 PMC2492627

[ref19] Izumi M. Tsunoda H. Suzuki Y. Makino A. Ishida H. (2012). RBCS1A and RBCS3B, two major members within the Arabidopsis RBCS multigene family, function to yield sufficient Rubisco content for leaf photosynthetic capacity. J. Exp. Bot. 63, 2159–2170. doi: 10.1093/jxb/err434, PMID: 22223809 PMC3295403

[ref20] Jiang Y. Liang G. Yang S. Yu D. (2014). Arabidopsis WRKY57 functions as a node of convergence for Jasmonic acid- and auxin-mediated signaling in jasmonic acid-induced leaf senescence. Plant Cell 26, 230–245. doi: 10.1105/tpc.113.117838, PMID: 24424094 PMC3963572

[ref21] Keech O. Pesquet E. Gutierrez L. Ahad A. Bellini C. Smith S. M.. (2010). Leaf senescence is accompanied by an early disruption of the microtubule network in Arabidopsis thaliana. Plant Physiol. 154, 1710–1720. doi: 10.1104/pp.110.163402, PMID: 20966154 PMC2996031

[ref22] Khan I. U. Akhtar A. Haris A. K. Dongwon B. Junghoon P.. (2020). WR/HDA9/ABI4 Complex Epigenetically Regulates ABA Dependent Drought Stress Tolerance in Arabidopsis. Front. Plant Sci. 11:623. doi: 10.3389/fpls.2020.00623, PMID: 32528497 PMC7266079

[ref23] Kim H. J. Nam H. G. Lim P. O. (2016). Regulatory network of NAC transcription factors in leaf senescence. Curr. Opin. Plant Biol. 33, 48–56. doi: 10.1016/j.pbi.2016.06.002, PMID: 27314623

[ref24] Kim Y. S. Sakuraba Y. Han S. H. Yoo S. C. Paek N. C. (2013). Mutation of the Arabidopsis NAC016 transcription factor delays leaf senescence. Plant Cell Physiol. 54, 1660–1672. doi: 10.1093/pcp/pct113, PMID: 23926065

[ref25] Kim J. H. Woo H. R. Kim J. Lim P. O. Lee I. C. Choi S. H.. (2009). Trifurcate feed-forward regulation of age-dependent cell death involving miR164 in Arabidopsis. Science 323, 1053–1057. doi: 10.1126/science.1166386, PMID: 19229035

[ref26] Lee J. H. Terzaghi W. Gusmaroli G. Charron J. B. F. Yoon H. J. Chen H.. (2008). Characterization of Arabidopsis and rice DWD proteins and their roles as substrate receptors for CUL4-RING E3 ubiquitin ligases. Plant Cell 20, 152–167. doi: 10.1105/tpc.107.055418, PMID: 18223036 PMC2254929

[ref27] Li Z. Jinying P. Xing W. Hongwei G. (2013). ETHYLENE-INSENSITIVE3 is a senescence-associated gene That accelerates age-dependent leaf senescence by directly repressing miR164 transcription in Arabidopsis. Plant Cell 25: 3311–3328. doi: 10.1105/tpc.113.113340, PMID: 24064769 PMC3809534

[ref28] Li Z. Peng J. Wen X. Guo H. (2012). Gene network analysis and functional studies of senescence-associated genes reveal novel regulators of Arabidopsis leaf senescence. J. Integr. Plant Biol. 54, 526–539. doi: 10.1111/j.1744-7909.2012.01136.x, PMID: 22709441

[ref29] Li Z. Woo H. R. Guo H. (2018). Genetic redundancy of senescence-associated transcription factors in Arabidopsis. J. Exp. Bot. 69, 811–823. doi: 10.1093/jxb/erx345, PMID: 29309664

[ref300] Liu P. Zhang S. Zhou B. Luo X. Zhou X. Cai B.. (2019). The histone H3K4 demethylase JMJ16 represses leaf senescence in Arabidopsis. Plant Cell 31, 1–15. doi: 10.1105/tpc.18.00693, PMID: 30712008 PMC6447021

[ref30] Lim P. O. Hong G. N. (2007). Aging and senescence of the leaf organ. J. Plant Biol. 50, 291–300. doi: 10.1007/BF03030657

[ref31] Lim C. J. Junghoon P. Mingzhe S. Hee J. P. Mi S. C. Ki S. P.. (2020). The histone-modifying complex PWR/HOS15/HD2C epigenetically regulates 5 cold tolerance. Plant Physiol. 184, 1097–1111. doi: 10.1104/pp.20.00439, PMID: 32732349 PMC7536694

[ref32] Lim P. O. Kim H. J. Nam H. G. (2007a). Leaf senescence. Annu. Rev. Plant Biol. 58, 115–136. doi: 10.1146/annurev.arplant.57.032905.105316 17177638

[ref33] Lohman K. N. Gan S. John M. C. Amasino R. M. (1994). Molecular analysis of natural leaf senescence in *Arabidopsis thaliana*. Physiol. Plant. 92, 322–328. doi: 10.1111/j.1399-3054.1994.tb05343.x

[ref34] Mayer K. S. Chen X. Sanders D. Chen J. Jiang J. Nguyen P.. (2019). HDA9-PWR-HOS15 is a core histone deacetylase complex regulating transcription and development. Plant Physiol. 180, 342–355. doi: 10.1104/pp.18.01156, PMID: 30765479 PMC6501109

[ref35] Mayta M. L. Mohammad-Reza H. Néstor C. Anabella F. L. (2019). Leaf senescence: The chloroplast connection comes of age. Plan. Theory 8:495. doi: 10.3390/plants8110495 PMC6918220 31718069

[ref36] Oda-Yamamizo C. Mitsuda N. Sakamoto S. Ogawa D. Ohme-Takagi M. Ohmiya A. (2016). The NAC transcription factor ANAC046 is a positive regulator of chlorophyll degradation and senescence in Arabidopsis leaves. Sci. Rep. 6:23609. doi: 10.1038/srep23609, PMID: 27021284 PMC4810360

[ref37] Oh S. A. Lee S. Y. Chung I. K. Lee C. H. Nam H. G. (1996). A senescence-associated gene of Arabidopsis thaliana is distinctively regulated during natural and artificially induced leaf senescence. Plant Mol. Biol. 30, 739–754. doi: 10.1007/BF00019008, PMID: 8624406

[ref38] Park H. J. Baek D. Cha J. Y. Liao X. Kang S. H. McClung C. R.. (2019). HOS15 interacts with the histone deacetylase HDA9 and the evening complex to epigenetically regulate the floral activator GIGANTEA. Plant Cell 31, 37–51. doi: 10.1105/tpc.18.00721, PMID: 30606777 PMC6391688

[ref39] Park J. Lim C. J. Shen M. Park H. J. Cha J. Y. Iniesto E.. (2018a). Epigenetic switch from repressive to permissive chromatin in response to cold stress. Proc. Natl. Acad. Sci. U. S. A. 115, E5400–E5409. doi: 10.1073/pnas.1721241115, PMID: 29784800 PMC6003311

[ref40] Park J. Lim C. J. Khan I. U. Jan M. Khan H. A. Park H. J.. (2018b). Identification and molecular characterization of HOS15- interacting proteins in Arabidopsis thaliana. J. Plant. Biol. 61, 336–345. doi: 10.1007/s12374-018-0313-2

[ref41] Park J. H. Oh S. A. Kim Y. H. Woo H. R. Nam H. G. (1998). Differential expression of senescence-associated mRNAs during leaf senescence induced by different senescence-inducing factors in Arabidopsis. Plant Mol. Biol. 37, 445–454. doi: 10.1023/A:1005958300951 9617812

[ref43] Ren Y. Li Y. Jiang Y. Wu B. Miao Y. (2017). Phosphorylation of WHIRLY1 by CIPK14 shifts its localization and dual functions in Arabidopsis. Mol. Plant 10, 749–763. doi: 10.1016/j.molp.2017.03.011, PMID: 28412544

[ref44] Sakuraba Y. Jeong J. Kang M. Y. Kim J. Paek N. C. Choi G. (2014). Phytochrome-interacting transcription factors PIF4 and PIF5 induce leaf senescence in Arabidopsis. Nat. Commun. 5:4636. doi: 10.1038/ncomms5636, PMID: 25119965

[ref45] Sakuraba Y. Piao W. Lim J. H. Han S. H. Kim Y. S. An G.. (2015b). Rice ONAC106 inhibits leaf senescence and increases salt tolerance and tiller angle. Plant Cell Physiol. 56, 2325–2339. doi: 10.1093/pcp/pcv144, PMID: 26443376

[ref46] Saleh A. Alvarez-Venegas R. Avramova Z. (2008). An efficient chromatin Immunoprecipitation (ChIP) protocol for studying histone modifications in *Arabidopsis* plants. Nat. Protoc. 3, 1018–1025. doi: 10.1038/nprot.2008.66, PMID: 18536649

[ref47] Shen M. Chae J. L. Junghoon P. Jeong E. K. Dongwon B. Jaesung N.. (2020). HOS15 is a transcriptional corepressor of NPR1-mediated gene activation of plant immunity. PNAS 117, 30805–30815. doi: 10.1073/pnas.2016049117, PMID: 33199617 PMC7720166

[ref48] Smart C. M. (1994). Gene expression during leaf senescence. New Phytol. 126, 419–448. doi: 10.1111/j.1469-8137.1994.tb04243.x 33874468

[ref49] Song Y. Yang C. Gao S. Zhang W. Li L. Kuai B. (2014). Age-triggered and dark-induced leaf senescence require the bHLH transcription factors PIF3/4/5. Mol. Plant 7, 1776–1787. doi: 10.1093/mp/ssu109, PMID: 25296857 PMC4261840

[ref50] Sun G. Mei Y. Deng D. Xiong L. Sun L. Zhang X.. (2017). N-terminus-mediated degradation of ACS7 is negatively regulated by senescence signaling to allow optimal Ethylene production during leaf development in Arabidopsis. Front. Plant Sci. 8:2066. doi: 10.3389/fpls.2017.02066, PMID: 29270180 PMC5723933

[ref51] Takasaki H. Maruyama K. Takahashi F. Fujita M. Yoshida T. Nakashima K.. (2015). SNAC-as, stress-responsive NAC transcription factors, mediate ABA-inducible leaf senescence. Plant J. 84, 1114–1123. doi: 10.1111/tpj.13067, PMID: 26518251

[ref52] Trivellini A. Jibran R. Watson L. M. O’Donoghue E. M. Ferrante A. Sullivan K. L.. (2012). Carbon deprivation-driven transcriptome reprogramming in detached developmentally arresting Arabidopsis inflorescences. Plant Physiol. 160, 1357–1372. doi: 10.1104/pp.112.203083, PMID: 22930749 PMC3490613

[ref53] Verdin E. Ott M. (2015). 50 years of protein acetylation: from gene regulation to epigenetics, metabolism and beyond. Nat. Rev. Mol. Cell Biol. 16, 258–264. doi: 10.1038/nrm3931, PMID: 25549891

[ref54] Veronese P. Nakagami H. Bluhm B. Abuqamar S. Chen X. Salmeron J.. (2006). The membrane-anchored BOTRYTIS-INDUCED KINASE1 plays distinct roles in Arabidopsis resistance to necrotrophic and biotrophic pathogens. Plant Cell 18, 257–273. doi: 10.1105/tpc.105.035576, PMID: 16339855 PMC1323497

[ref55] Wang W. Ye R. Xin Y. Fang X. Li C. Shi H.. (2011a). An importin beta protein negatively regulates MicroRNA activity in Arabidopsis. Plant Cell 23, 3565–3576. doi: 10.1105/tpc.111.091058, PMID: 21984696 PMC3229135

[ref56] Wang Y. Nishimura M. T. Zhao T. Tang D. (2011b). ATG2, an autophagy-related protein, negatively affects powdery mildew resistance and mildew-induced cell death in Arabidopsis. Plant J. 68, 74–87. doi: 10.1111/j.1365-313X.2011.04669, PMID: 21645148

[ref57] Weaver L. M. Amasino R. M. (2001). Senescence is induced in individually darkened Arabidopsis leaves, but inhibited in whole darkened plants. Plant Physiol. 127, 876–886. doi: 10.1104/pp.010312, PMID: 11706170 PMC129259

[ref58] Weaver L. M. Gan S. Quirino B. Amasino R. M. (1998). A comparison of the expression patterns of several senescence-associated genes in response to stress and hormone treatment. Plant Mol. Biol. 37, 455–469. doi: 10.1023/A:1005934428906 9617813

[ref59] Weaver L. M. Himelblau E. Amasino R. M. (1997). “Leaf senescence: gene expression and regulation,” in Genetic Engineering. *Vol*. 19. ed. Setlow J. K. (New York: Plenum Press), 215–234.

[ref60] Zentgraf U. Jobst J. Kolb D. Rentsch D. (2004). Senescence-related gene expression profiles of rosette leaves of Arabidopsis thaliana: leaf age versus plant age. Plant Biol. 6, 178–183. doi: 10.1055/s-2004-815735, PMID: 15045669

[ref61] Zhang Y. Liu Z. Chen Y. He J. X. Bi Y. (2015). PHYTOCHROME- INTERACTING FACTOR 5 (PIF5) positively regulates dark-induced senescence and chlorophyll degradation in Arabidopsis. Plant Sci. 237, 57–68. doi: 10.1016/j.plantsci.2015.05.010, PMID: 26089152

[ref62] Zhu J. Jeong J. C. Zhu Y. Sokolchik I. Miyazaki S. Zhu J. K.. (2008). Involvement of Arabidopsis HOS15 in histone deacetylation and cold tolerance. Proc. Natl. Acad. Sci. U. S. A. 105, 4945–4950. doi: 10.1073/pnas.0801029105, PMID: 18356294 PMC2290775

