# Supplementary material for: The Transcriptional Corepressor HOS15 Mediates Dark-Induced Leaf Senescence in Arabidopsis
Source: Front Plant Sci. 2022 Feb 25;13:828264. doi: 10.3389/fpls.2022.828264 (PMC8914473; doi:10.3389/fpls.2022.828264)
Supplement: Supplementary file 1 [file Data_Sheet_1.PDF]

Supplementary Material

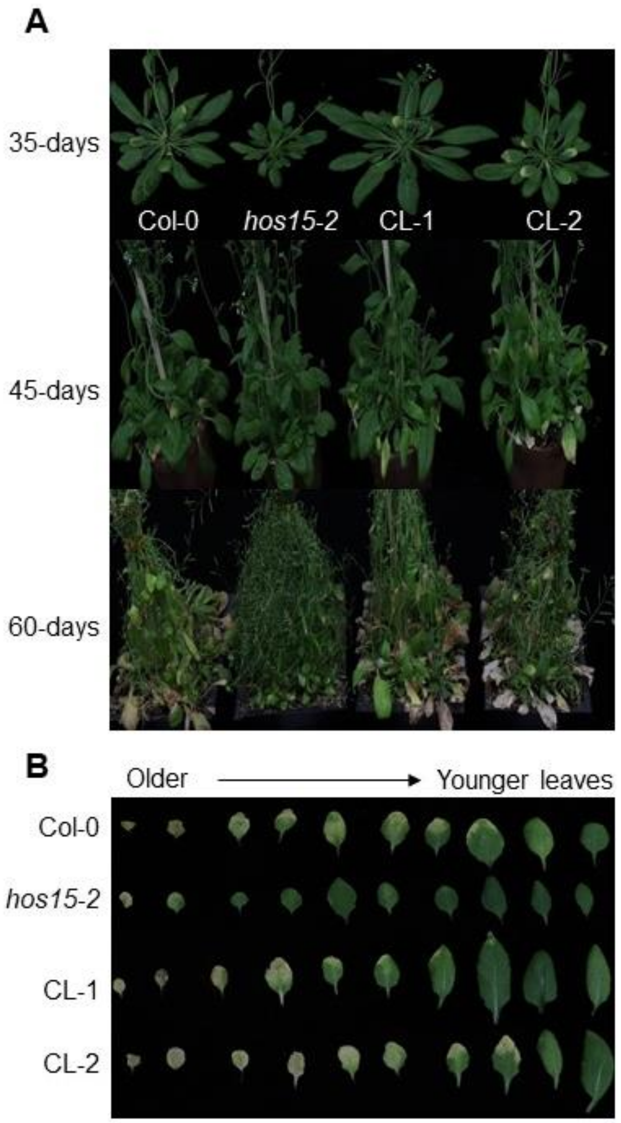

Supplementary Figure 1. (ref. to Figure 1A/B). HOS15 mediates senescence in an age-dependent manner. (A) HOS15 positively regulates plant senescence in an age-dependent manner. Phenotypic analysis of Col-0 (WT), *hos15-2,* and two complemented lines (HOS15::HOS15/*hos15-2*), CL-1 and CL-2, after 35, 45, and 60 days of germination. The seeds of Col-0, *hos15-2*, CL-1, and CL-2 were grown on MS medium for 12 days and then transferred to soil. (B) Rosette leaf phenotypes of Col-0, hos15-2, CL-1, and CL-2 plants after 45 days of germination. The rosette leaves (3rd-12th leaves) were arranged according to their age (from older to younger leaves).

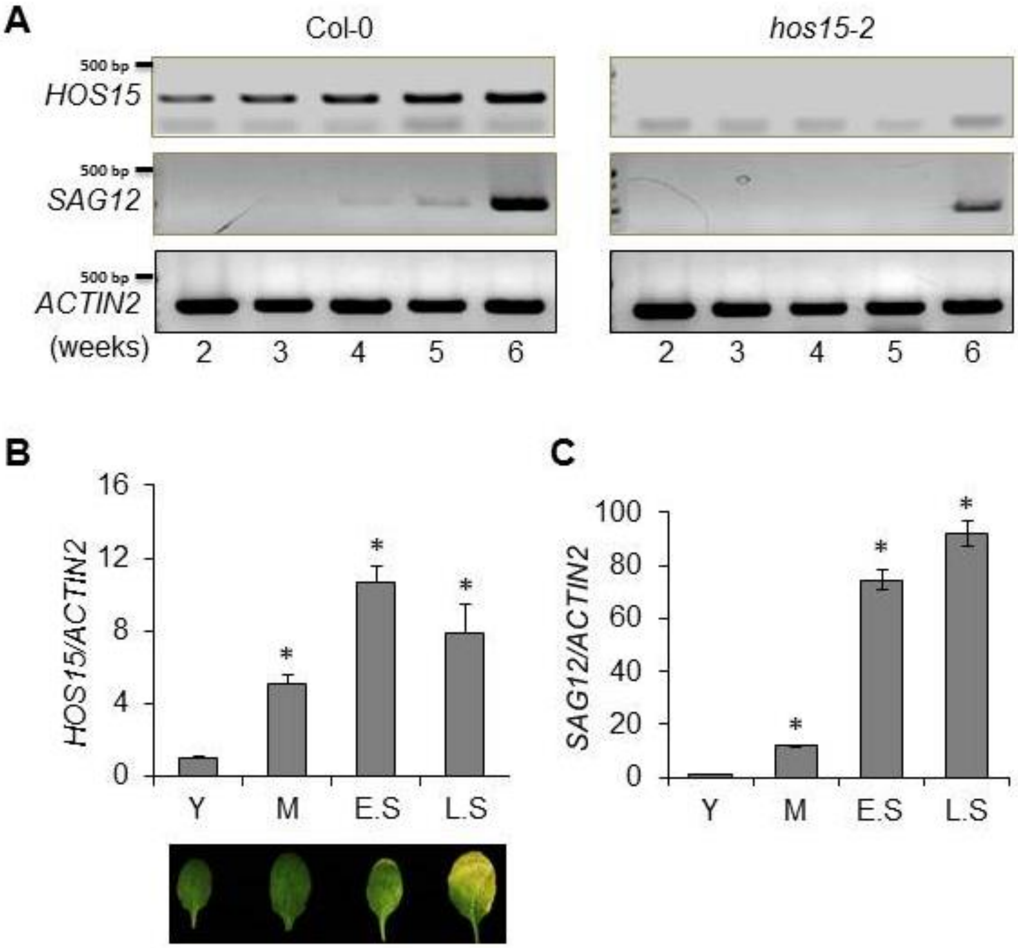

Supplementary Figure 2. (ref. to Figure 1A,B). HOS15 mediates senescence in an age-dependent manner. (A) The mRNA expression analysis of HOS15 and SAG15 genes in Col-0 and *hos15-2* plants in an age-dependent manner. Seeds of Col-0 (WT) and loss-of-function HOS15 (*hos15-2*) plants were grown on MS medium, and 12-days-old seedlings were transferred to soil. The 3rd and 4th rosette leaves of both plants were sampled according to the mentioned ages (2-6-weeks-old 3rd and 4th rosette leaves) for RNA extraction and cDNA synthesis. Transcript levels of HOS15 and SAG12 were analyzed using qRT-PCR. ACTIN2 was used as the internal control. (B and C) The expression of HOS15 and SAG12 was analyzed by qRT-PCR according to their age (Y=Young, M=Mature, ES=Early Senescence, and LS=Late Senescence) in Col-0 plants. ACTIN2 was used as the normalization control. Error bars represent the SD of three independent biological replicates. Significant differences were tested using Student's *t*-test (*P < 0.05).

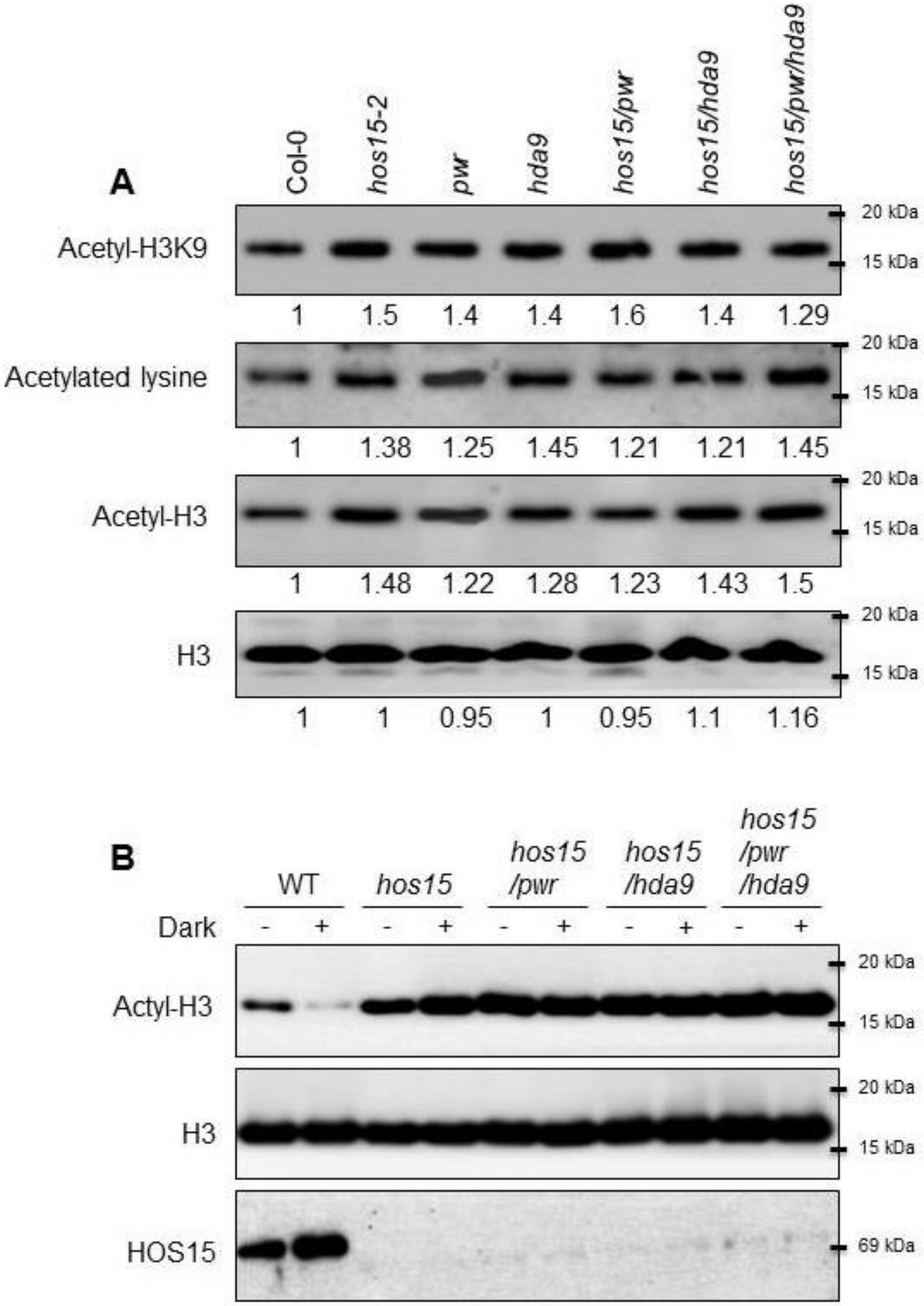

Supplementary Figure 3. The HOS15-PWR-HDA9 complex participates equally in histone deacetylation during senescence. (A) Comparative analysis of H3 acetylation status during early senescence. The nuclear proteins of Col-0 (WT), *hos15-2, pwr,hda9, hos15pwr, hos15hda9, and hos15pwrhda9* mutants were extracted from early senescent leaves. Anti-H3, Anti-AcH3, Anit-H3K9, anti-AcK antibodies were used for H3 analysis. Anti-H3 antibody was used as the loading control. Each experiment was repeated three times with similar results. In the bottom of each blot band intensities have been calculated using image J software. **(B)** HOS15 together with HDA9 and PWR regulates dark-induced senescence by affecting H3 acetylation status. 12-day-old seedlings of Col-0

and *hos15-2* plants were treated with 4 days of darkness, and then nuclear proteins were extracted. H3, AcH3 and HOS15 antibodies were used for WB. H3 was used as the loading control.

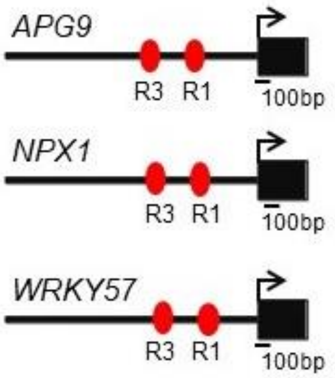

Supplementary Figure 4. HOS15 associates with APG9, NPX1 and WRKY57 promoter regions. (A) schematic representation of the APG9, NPX1 and WRKY57 promoter and amplicon regions (R1 and R3) for Chip-qRT-PCR. These regions are modified from Chen et al. 2016, mentioned as P1, P2 and P3 (here P2=R1 and P3=R3). Primer positions are indicated with R1 and R3. The arrow indicates the transcription start site.

Supplementary Table 1. Primers used in the study

PRIMER SEQUENCE	
SAG12-F	CAGCTGCGGATGTTGTTG
SAG12-R	CCACTTTCTCCCCATTTTG
SAG29-F	CTGTTTTCGCTGCCCCTC
SAG29-R	ACAGCCCTAGTACGAATCCCAC
ORE1-F	ACGTGCCGATGGTACAAAGGTTC
ORE1-R	TCTTGGTCGGAGAAGCAGGTCAC
CAB1-F	GCAAGGACCCGTGAACTAGAA
CAB1-R	TCCGAACTTGACTCCGTTTC
RCBS1A-F	CGCTCCTTTCAACGGACTTA
RCBS1A-R	AGTAATGTCGTTAGCCTTGC
ACTIN2-F	AACCACTATGTTCTCAGGCATCG
ACTIN2-R	CCTGGACCTGCCTCATCATACT
HOS15-F	GATGGCCAAGCAAGAATCTG
HOS15-R	TCCTGTAGGGCTCCATCTGA
WRKY57- R1-F	CACTGCACTTTAACGGGTTTCAAAGT
tssWRKY57- R1-R	GATCGGCGAGAGAAGTAGTGATAAGAG
tssNPX1- R1-F	AGCTCTCGATCTAGGGTTTTCC
tssNPX1- R1-R	CTCAACGGCAATTGGCAAAAA

tssAPG9- R1-F	AGAAGAGGAAGAGAACTCGTGAT
tssAPG9- R1-R	TTGATGCTTTGGAGTTTGGAGT
WRKY57- R3-F	GTCGGTGGCAGTTGGAGTAA
WRKY57- R3-R	ACGTGAGACGCTTTTTGACC
ACTIN7 F CHIP	CGTTTCGCTTTCCTTAGTGTTA
ACTIN7 R CHIP	AGCGAACGGATCTAGAGCTC
APG9- R3-F	AGGTGATTTGCATTGTGGATGCT
APG9- R3-R	CGATTGGCCAAAACAGCCG
NPX1- R3-F	TCCACCAGGAAATCAGTTCCATA
NPX1-R3-R	ATCGGTGCTACTTCGAAAGGG
